# Cerebral mitochondrial electron transport chain dysfunction in multiple system atrophy and Parkinson’s disease

**DOI:** 10.1038/s41598-019-42902-7

**Published:** 2019-04-25

**Authors:** Sandrine C. Foti, Iain Hargreaves, Stephanie Carrington, Aoife P. Kiely, Henry Houlden, Janice L. Holton

**Affiliations:** 1 Queen Square Brain Bank for Neurological Disorders, Department of Clinical and Movement Neurosciences, UCL Queen Square Institute of Neurology, University College London, London, UK; 2 Department of Neurodegenerative Diseases, UCL Queen Square Institute of Neurology, University College London, London, UK; 3 UCL Institute of Neurology, National Hospital for Neurology and Neurosurgery, London, WC1N 3BG United Kingdom; 4 Liverpool John Moores University, School of Pharmacy and Biomedical Sciences, Liverpool, L3 3AF United Kingdom

**Keywords:** Parkinson's disease, Molecular neuroscience

## Abstract

Multiple system atrophy (MSA) is a neurodegenerative disease characterised by glial cytoplasmic inclusions (GCIs), containing α-synuclein. Mutated COQ2, encoding an enzyme essential for co-enzyme Q10 (CoQ10) biosynthesis, has been associated with MSA. CoQ10 is an electron carrier in the mitochondrial electron transport chain (ETC) and antioxidant. It has been shown to be deficient in MSA brain tissue, thus implicating mitochondrial dysfunction in MSA. To investigate mitochondrial dysfunction in MSA further we examined ETC activity in MSA and control brain tissue, compared with Parkinson’s disease (PD) where mitochondrial dysfunction is known to be important. Using cerebellar and occipital white matter ETC complex I, II/III and IV activities were measured spectrophotometrically, selected individual components of the ETC were assessed by immunoblotting and cellular complex IV activity was analysed by enzyme histochemistry. We show decreased complex II/III activity with increased complex I and IV activity in MSA cerebellar white matter. This corresponds with the deficit in CoQ10 previously described in MSA and reflects the high regional pathological burden of GCIs. This study highlights mitochondrial dysfunction in MSA pathogenesis, suggests an influence on selective regional vulnerability to disease and points to shared disease mechanisms in α-synucleinopathies.

## Introduction

Multiple system atrophy (MSA) is a progressive and debilitating neurodegenerative disease presenting with combinations of clinical features including cerebellar ataxia (MSA-C), parkinsonism (MSA-P), autonomic dysfunction and pyramidal signs^1^. Neuropathological examination shows neurodegeneration in different brain regions resulting in neuropathological MSA subtypes of olivopontocerebellar atrophy (OPCA), striatonigral degeneration (SND) or a combination of these two (SND = OPCA or mixed)^2,3^. MSA is an α-synucleinopathy, a group of disorders which also includes Parkinson’s disease (PD) and dementia with Lewy bodies (DLB). In contrast with the neuronal Lewy body inclusions featured in PD and DLB, the neuropathological hallmark of MSA is the widespread presence of glial cytoplasmic inclusions (GCIs) composed of aggregated α-synuclein in oligodendrocytes^4–7^. This feature has led to MSA being considered as an oligodendrogliopathy^8^.

Although MSA is regarded as a sporadic disease, genetic factors have been implicated in the aetiology of this disorder. These include: copy number loss of C-terminal Src homology 2 Adapter Protein 2 (SHC2)^9^, SNCA gene (synuclein alpha) single-nucleotide polymorphisms^10,11^ and mutation in the *CoQ2* gene which encodes for 4 hydroxbenzoate polyprenyltransferase (CoQ2), an enzyme involved in coenzyme Q10 (CoQ10) biosynthesis^12,13^. Interestingly, a genome-wide association study (GWAS) in MSA identified single nucleotide polymorphisms (SNPs) in four genes which did not include *SNCA* or *CoQ2*^14^.

The candidate genes potentially predisposing individuals to developing MSA^15^ have mainly been linked to neuroinflammation. Although, this appears to be additionally influenced by the geographical distribution of patients^16–18^. Differentially expressed genes in MSA have been shown to be involved in mitochondrial function^19^. Furthermore, a Japanese study revealed that there is an increased risk of MSA in multiplex families when they have a functionally impaired variant of *CoQ2*^12^. CoQ2 is important for the synthesis of CoQ10 which is a powerful cellular antioxidant and an electron carrier, transporting electrons derived from complex I and II to complex III in the mitochondrial electron transport chain (ETC). CoQ10 levels were measured in MSA patients who were homozygous for a particular *CoQ2* variant (M128V-V393A) and found to be significantly reduced when compared to controls^12^. Deficiencies in CoQ10 levels were also identified in post-mortem pathological confirmed MSA cases with no *CoQ2* variants^20,21^. Studies in PD patients have also reported a reduction in CoQ10 levels in mitochondria in the blood and platelets when compared to age/gender matched control subjects^22,23^. In addition, a deficit in CoQ10 status has also been reported in cerebral cortex of PD patients^24^.

The function of the mitochondrial ETC can be influenced by many biological processes, including oxidative stress which occurs when there is an inbalance between reactive oxygen species (ROS) generation and cellular antioxidant status^25^. In the presence of high levels of ROS, oxidative stress can be induced, leading to deleterious changes in mitochondrial function^26^ as well as inducing an innate immune response^27^ and causing a diminution in cellular antioxidant defences. The observed decrease in cerebellar CoQ10 in MSA suggests that the function of the ETC may be disturbed in this disease^12,20,21^.

The ETC defects observed in Alzheimer’s disease (AD), PD and MSA have been attributed to somatic mitochondrial (mt) DNA mutations^28,29^ where high levels of mtDNA deletion or depletion affect the activity as well as the subunit expression of mtDNA encoded ETC complex subunits (complex I, III, IV and V)^30^. Complex II is encoded entirely by nuclear DNA and therefore is spared in conditions associated with mtDNA mutations. The most prominent mechanism of mtDNA impairment is via ROS generated by the ETC^31^. ROS are produced by the ETC, principally at complex I and III but there is evidence that complex II also contributes to the ROS pool^32–34^.

The aim of this study was to investigate whether the changes in CoQ10 levels previously described in MSA are associated with ETC dysfunction. To do this we used spectrophotometric enzyme assays to measure the activities of ETC complexes I, II/III and IV, immunoblotting to determine the protein expression of selected individual components of these complexes and enzyme histochemistry to determine complex IV activity at the cellular level. The white matter from cerebellum and occipital lobe were used to represent brain regions respectively highly or mildly affected in MSA. Both regions may show minimal α-synuclein pathology in the form of Lewy neurites in PD (Supplementary Fig. 1). To minimise any possible influence of neuropathological subtype in MSA we selected a cohort of mixed MSA cases and compared these with PD as an α-synculeinopathy disease control and neurologically normal controls using frozen post-mortem brain tissue.

## Results

### Electron transport chain complex activity

**Figure 1:**
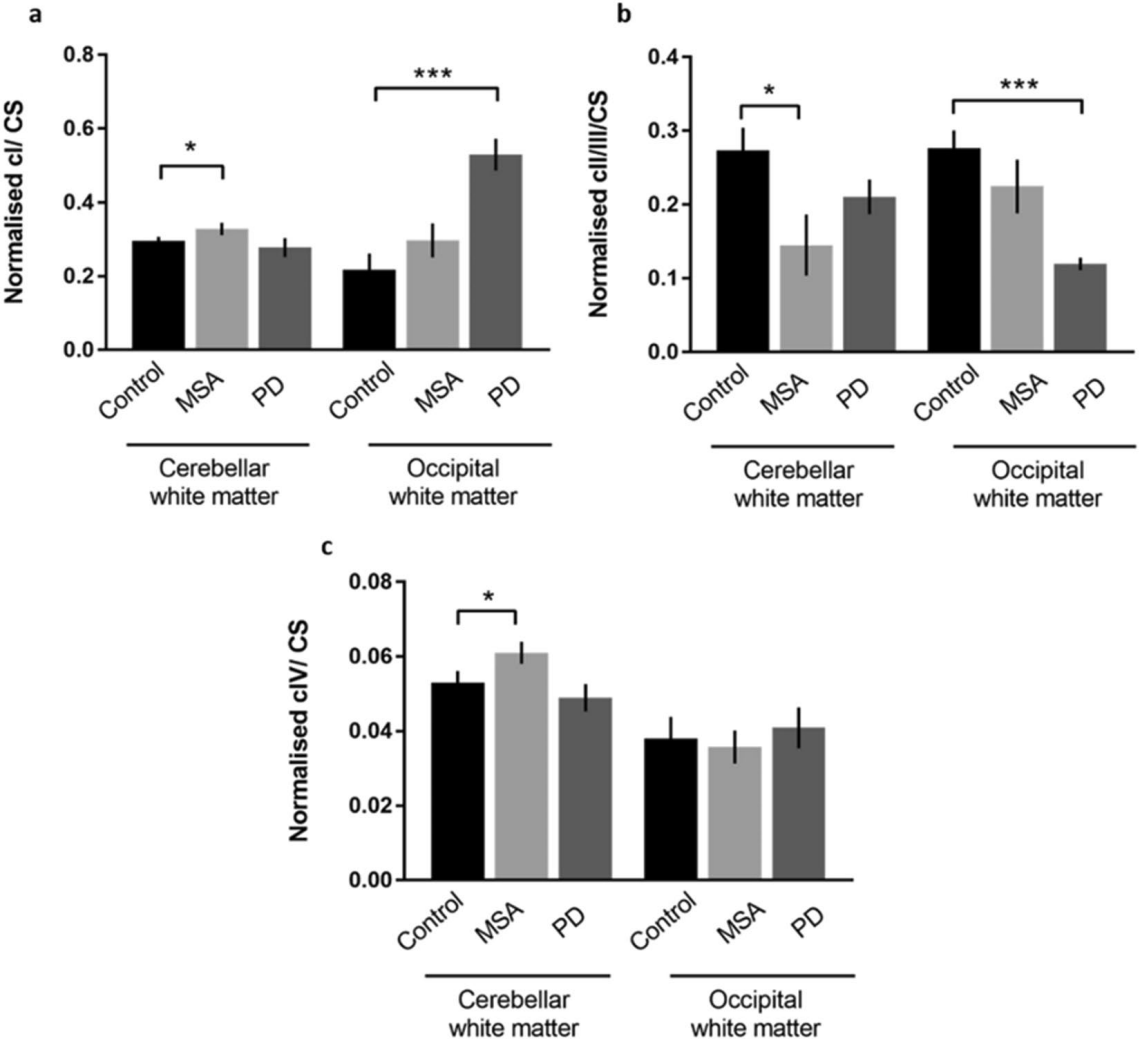
Activity of elements of the ETC in MSA and PD. Measuring ETC activity in post-mortem tissue from MSA and PD cases revealed dysregulation of activity when compared to controls in cerebellar and occipital white matter. Complex I activity increased in MSA cerebellar white matter and PD occipital white matter when compared to control cases **(a)**. However downstream to complex I, complex II/III activity was significantly reduced in both MSA cerebellar white matter and PD occipital white matter **(b)**. Significant changes in complex IV activity were found only in the cerebellar white matter where activity in MSA was increased compared to control **(c)**. Significance was set at p < 0.05 (*), p < 0.01 (**) and p < 0.001 (***).

The activity of ETC complexes I, II/III and IV was measured in the cerebellar white matter, a region with large numbers of α-synuclein positive GCIs^4,35^ and compared with the occipital white matter, where GCIs are sparse in MSA (Supplementary Fig. 1)^36,37^. Cases of MSA and PD, chosen as an α-synucleinopathy disease control in which there is minimal α-synuclein pathology in the white matter in these regions, were compared with normal controls. All values are presented as the ratio between enzyme activity and citrate synthase (CS) activity to control for mitochondrial mass (Fig. 1). The levels of complex I activity revealed a small but significant increase between MSA and controls in the cerebellar white matter (p = 0.041). Increased complex I activity was also observed in the occipital white matter in PD cases (p = 0.0001) when compared to control cases (Fig. 1a). Complex II/III activity was reduced in both brain regions compared with control and this was significant in the cerebellar white matter in MSA cases (p = 0.0242) and in the occipital white matter in PD (p = 0.0002) (Fig. 1b). Complex IV activity showed changes in the cerebellar white matter where a small augmentation in the activity was measured in MSA when compared to controls (p = 0.0404). No significant difference was observed in the complex IV activity in PD compared with controls (Fig. 1c).

### Mitochondrial ETC complex protein subunit expression

**Figure 2:**
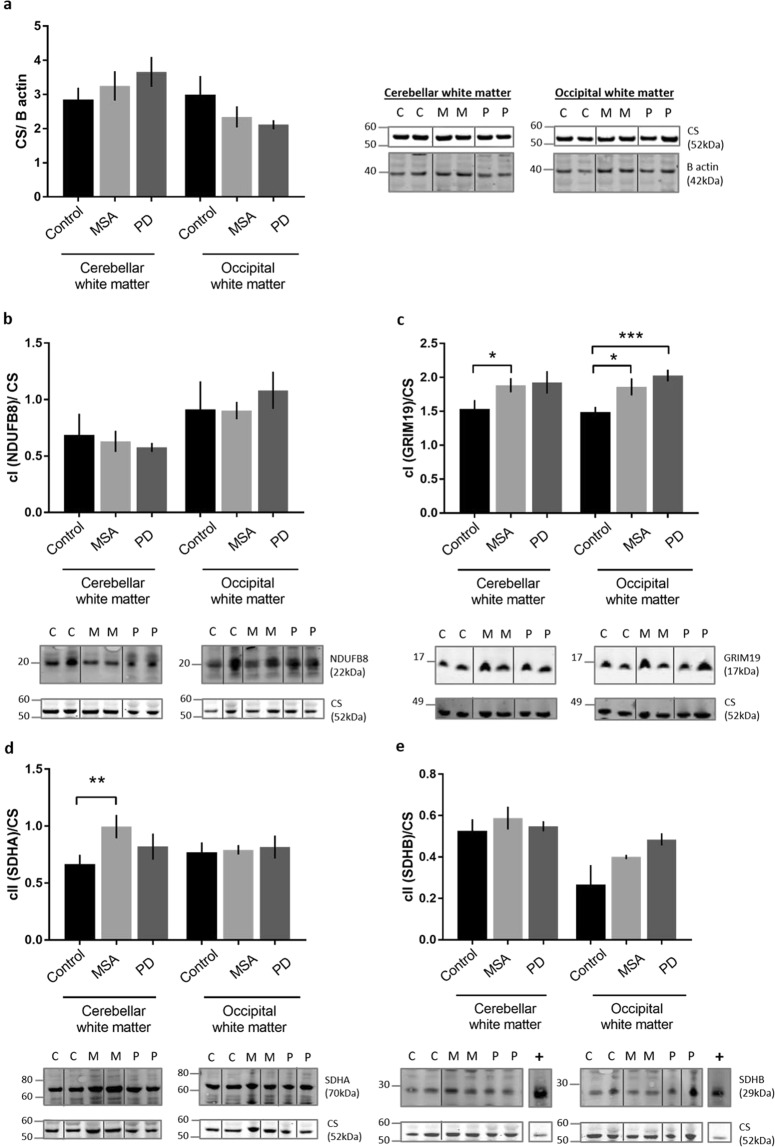
Citrate synthase and complex I and II subunit expression in MSA and PD. The protein expression levels of CS, a mitochondrial biomass marker as well as specific complex I and complex II subunits were measured using western blotting in control, MSA and PD cases. Representative western blots are shown under each bar graph. Each band chosen originates from the same gel. The mitochondrial biomass of all groups remained indistinguishable which was reflected by the unchanged CS protein levels after normalisation to the house keeping protein, β actin **(a)**. Western blotting for complex I, NDUFB8 subunit showed no changes across all groups and brain regions **(b)**. Complex I subunit GRIM19 showed an increase in MSA cerebellar and occipital white matter, as well as an increase in PD occipital white matter **(c)**. The complex II subunit SDHA showed a significant increase in expression in MSA cerebellar white matter when compared to controls **(d)**. The SDHB subunit remained consistent between all groups **(e)**. Immunoblot abbreviations (C = control, M = MSA, P = PD, + = positive control, human heart lysate). Significance was set at p < 0.05 (*), p < 0.01 (**) and p < 0.001 (***). Full length gels are demonstrated in the Supplementary Figs 2, 3, 5 and 6.

In order to address whether the changes observed in ETC activity may be related to changes in protein expression of components of the ETC complexes, immunoblotting was carried on whole homogenate from cerebellar and occipital white matter from control, MSA and PD cases. The expression of the mitochondrial biomass marker, CS was normalised to the house keeping protein, β-actin, and showed no significant changes in MSA or PD compared with control in either region (Fig. 2a). Selected individual components of complexes I–IV of the ETC were quantitated by normalising to CS. The nuclear encoded subunit NADH dehydrogenase [ubiquinone] 1 beta subcomplex subunit 8 (NDUFB8) of complex I expression levels remained unchanged in PD and MSA in each brain region (Fig. 2b). A complex I accessory subunit known as GRIM19 (Genes associated with Retinoid–IFN-induced Mortality-19), or NDUFA13, showed an increase in expression in cerebellar and occipital white matter in MSA as well as in the PD occipital matter (Fig. 2c; p = 0.0256, 0.0418, 0.001 respectively). The two subunits A and B from complex II, succinate dehydrogenase complex flavoprotein subunit A (SDHA/B) were measured and an increase in SDHA protein expression was found in the cerebellar white matter in MSA cases when compared to controls (Fig. 2d and e; p = 0.0164).

**Figure 3:**
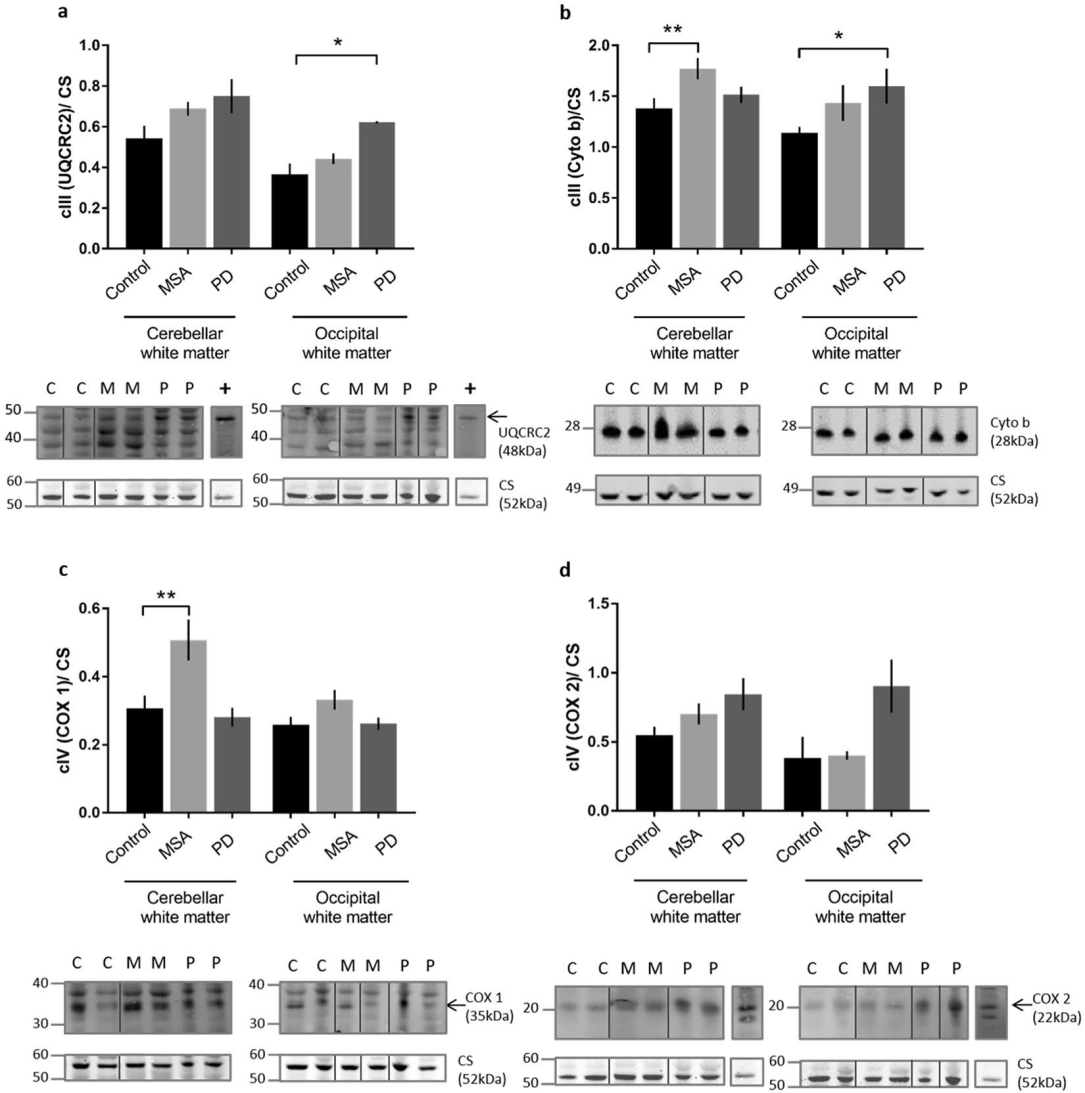
Complex III and IV subunit expression in MSA and PD. Western blotting of two subunits from complex III and from complex IV were performed in all groups. A representative western blot is shown below each bar graph. Each band chosen originates from the same gel. The only significant change seen in the UQCRC2 subunit of complex III was in PD occipital white matter **(a)**. Whereas cytochrome c, another complex III subunit not only showed an increase in PD occipital white matter but also in MSA white matter **(b)**. When the two core subunits of cytochrome c oxidase (complex IV) were visualised, only COX 1 showed significant changes. An upregulation in protein expression was observed in the cerebellar white matter of MSA cases **(c)**. The protein levels of COX 2 subunit remained uniform in all groups and brain regions **(d)**. Immunoblot abbreviations (C = control, M = MSA, P = PD, + = positive control, human heart lysate). Significance was set at p < 0.05 (*), p < 0.01 (**) and p < 0.001 (***). Full length gels are demonstrated in the Supplementary Figs 2, 4 and 7.

To investigate complex III, which is composed of 11 protein subunits we measured the level of the nuclear encoded subunit ubiquinol-cytochrome c reductase core protein 2 (UQCRC2), a core structural component^38,39^. The cerebellar white matter showed no significant changes in protein expression in either MSA or PD. An increase in total UQCRC2 expression was observed in the PD occipital cortex when compared to control (Fig. 3a; p = 0.0159). The mitochondrial-encoded cytochrome b complex III subunit showed significant increases in MSA cerebellar white matter as well as PD occipital white matter (Fig. 3b; p = 0.036, 0.01). Complex IV has 14 subunits called cytochrome c oxidase (COX) where the large core catalytic subunits COX 1, COX 2 and COX 3 (also known as MT-CO1, MT-CO2 and MT-CO3 respectively) are encoded by mitochondrial DNA^40,41^. We measured the protein levels of COX 1 and COX 2. COX 1 subunit showed elevated levels of expression in MSA cerebellar white matter when compared to control (Fig. 3c; p = 0.02). When examining the COX 2 subunit of complex IV there was a trend towards increased protein expression in the cerebellar white matter in PD and MSA but this was not statistically significant (Fig. 3d).

### Histochemical analysis of cytochrome c oxidase (complex IV) activity

**Figure 4:**
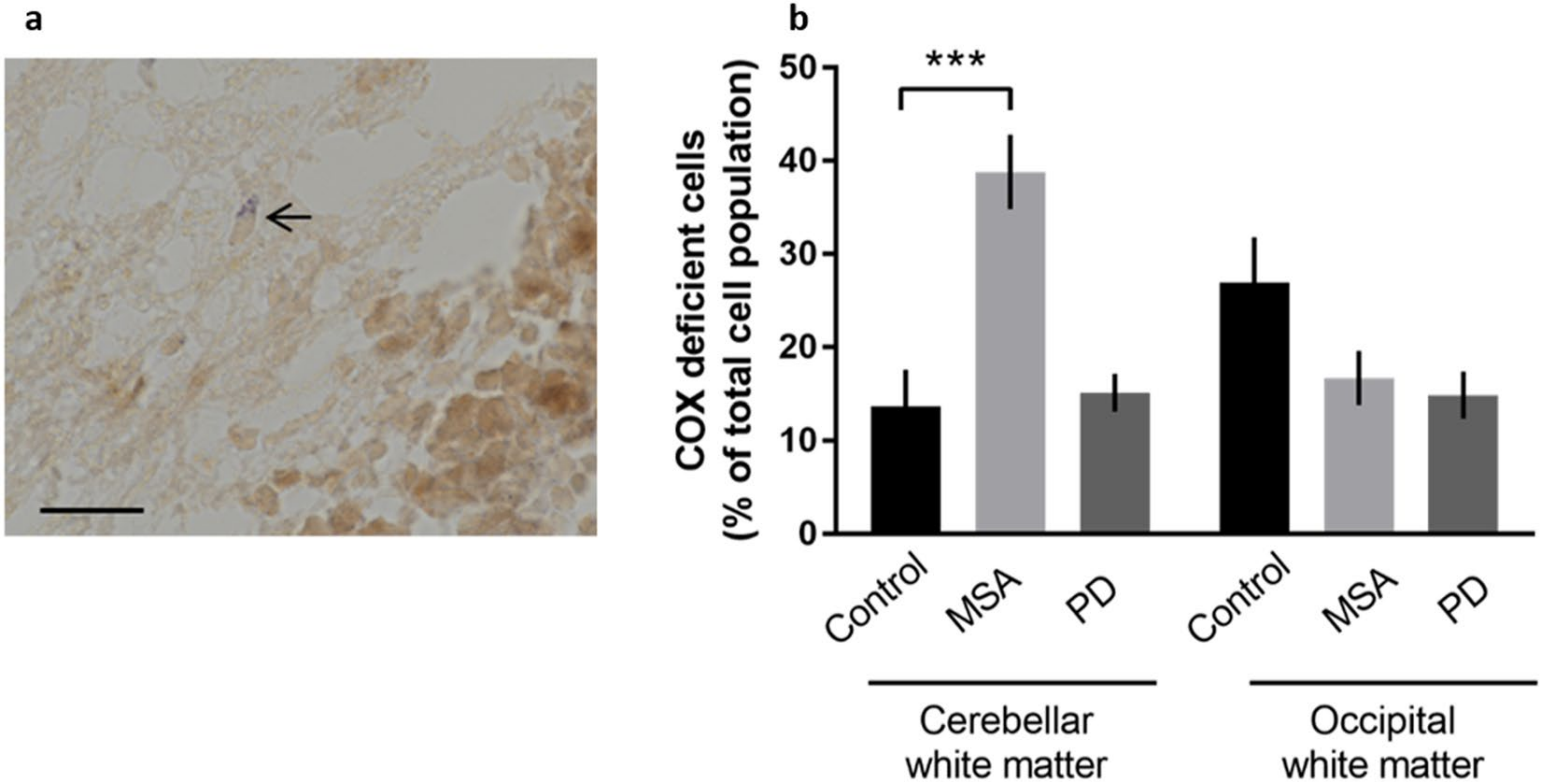
Complex IV activity measured using immunohistochemical technique in MSA and PD. COX/SDH histochemistry revealed differences in the number of COX deficient (blue) cells in MSA cerebellar white matter when compared to controls. The cytoplasm of a small number of glial cells in the cerebellar white matter was stained blue (arrow) reflecting intact SDH activity in the absence of COX activity **(a)**. Quantitation of the percentage of COX deficient cells revealed that these are more numerous in the cerebellar white matter in MSA than in controls (p < 0.0001) **(b)**. No differences were observed in the occipital white matter. Bar in A represents 20 µm. [n = 3 cases, 2 sections per case, 8 regions of interest per section analysed]. Significance was set at p < 0.05 (*), p < 0.01 (**) and p < 0.001 (***).

Sequential histochemical staining of frozen tissue sections for ETC cytochrome c oxidase (COX) and succinate dehydrogenase (SDH) permits the demonstration of cells with reduced complex IV activity^42^. In this assay, the individual cells which are COX-deficient are stained blue, reflecting SDH activity in the absence of COX activity while those with normal COX activity will be stained brown (Fig. 4a). We found an increased proportion of COX negative cells in the cerebellar white matter in MSA compared with controls (Fig. 4b; p < 0.0001). No difference was observed between the groups in the occipital white matter.

Table 1 summarises the results obtained throughout this study, including ETC activity and protein expression levels as well as COX/SDH immunohistochemical analysis.

**Table 1:** Summary of results.

	Cerebellar white matter		Occipital white matter	
	MSA	PD	MSA	PD
**Activity of components of the mitochondrial ETC**				
Complex I	Increased p = 0.041*	Unchanged	Unchanged	Increased P = 0.0001***
Complex II/III	Decreased P = 0.0242*	Unchanged	Unchanged	Decreased P = 0.0002***
Complex IV	Increased P = 0.0404*	Unchanged	Unchanged	Unchanged
**Mitochondrial mass**				
Citrate synthase protein expression	Unchanged	Unchanged	Unchanged	Unchanged
**Protein expression of selected subunits of components of the mitochondrial ETC**				
Complex I NDUFB8	Unchanged	Unchanged	Unchanged	Unchanged
Complex I GRIM19	Increased p = 0.0164*	Unchanged	Increased p = 0.0418*	Increased p = 0.001***
Complex II SDHA	Increased P = 0.0164**	Unchanged	Unchanged	Unchanged
Complex II SDHB	Unchanged	Unchanged	Unchanged	unchanged
Complex III UQCRC2	Unchanged	Unchanged	Unchanged	Increased P = 0.0159**
Complex III Cytochrome b	Increased p = 0.01**	Unchanged	Unchanged	Increased p = 0.036*
Complex IV COX1	Increased P = 0.02*	Unchanged	Unchanged	Unchanged
Complex IV COX2	Unchanged	Unchanged	Unchanged	Unchanged
**Cytochrome oxidase deficient cells identified by enzyme histochemistry**				
% COX negative cells	Increased p < 0.0001***	Unchanged	Unchanged	Unchanged

## Discussion

Altered mitochondrial function secondary to alterations in CoQ2 and CoQ10 has been proposed to play a role in the pathogenesis of MSA^12,20,21^. In view of the reduction in CoQ10 levels shown previously, we tested the hypothesis that this would result in disturbed mitochondrial function, manifesting as alterations in the ETC. We observed a significant reduction in ETC complex II/III activity in the cerebellar white matter of MSA cases (Fig. 1b). We also found an increase in both complex I and complex IV activities (Fig. 1a and c). These changes were restricted to the cerebellar white matter while the occipital cortex was unaffected reflecting the relative vulnerabilities of these regions to pathological change in MSA (Supplementary Fig. 1). Furthermore, these alterations in the activity of components of the ETC were not due to a reduction in mitochondrial mass, as illustrated by the unchanged citrate synthase protein levels (Fig. 2a). Changes in ETC activity did not consistently correlate with protein expression levels of individual components of the protein complexes of the ETC (Figs 2 and 3).

Understanding the relationship between the alterations observed in the ETC complex activities in MSA cerebellar white matter is important. The increase in complex I activity that we observed in this region could indicate a compensatory mechanism in response to the downstream reduction in complex II/III activity (Fig. 1a and b)^43^. Complexes I and III are the major source for ROS production, and when certain sites in these complexes are partially or fully impaired an increase in ROS production occurs^32^. A reduction in the ROS scavenger CoQ10, can lead to further increase in ROS levels which will expose cells to oxidative stress. As a consequence of this, conditions associated with CoQ10 deficiency, as has been observed in MSA, tend to have reduced activity of complex II/III as we have now demonstrated in MSA^20,21,44^.

Complex IV activity was also found to be elevated in MSA cerebellar white matter (Fig. 1c). Upregulation of complex IV activity has been observed in a range of physiological conditions. Complex IV mRNA and protein levels are increased by the free radical nitric oxide (NO)^45^. NO can be cytotoxic under certain conditions but may also act as an intracellular messenger and can induce the transcription of complex IV. In this context, sepsis leads to excess levels of reactive oxygen and nitrogen species and is associated with an increase in complex IV activity^46,47^. In addition to biochemical evaluation of ETC activity we also assessed this at the cellular level using the sequential COX/SDH histochemical assay. At a cellular level we found an increase in COX-deficient cells, representing glia, in MSA cerebellar white matter (Fig. 4). This demonstrates a complex situation in which individual cells may show decreased complex IV activity, while at the bulk tissue level there is an overall increase in activity which is likely to be related to oxidative stress. This situation is similar to that described in muscle disease where, despite the presence of individual cytochrome oxidase deficient muscle fibres identified by enzyme histochemistry, altered enzyme activity in muscle homogenate may not be detected^48^. It is also analogous to the finding that individual neurons show variable expression of components of the ETC complexes at the immunohistochemical level but this may not always be reflected in the enzyme activity assessed in tissue homogenates^49^. A previous study investigating ETC complex activity in cerebellar tissue from MSA cases did not demonstrate any changes in complex I + III, complex II + III or complex IV^21^. These results may differ from those in our study for reasons of case selection and tissue preparation. The MSA cohort studied by Barca *et al*.^21^, contained a combination of different MSA subtypes without apparent dissection of white matter, while in contrast, we restricted our study to mixed MSA cases with tissue enriched for white matter. Using mixed MSA only minimises any influence of neuropathological subtype. The use of post-mortem tissue to measure activity of the ETC and to determine loss of complex IV activity at the cellular level may be questioned in view of the potential for this to be influenced by post-mortem delay. However, it has previously been shown that ETC activity in the brain is not influenced by post-mortem delay^47^. Furthermore, these techniques have been employed in a number of studies^21,50–52^.

To probe the mechanisms underlying the observed alterations in ETC activity we investigated the protein expression of citrate synthase and selected components of the ETC complexes using immunoblotting. First we showed that there were no changes in the protein level of citrate synthase indicating that any changes were not secondary to alterations in the mitochondrial biomass (Fig. 2a). We had found that complex IV activity in MSA cerebellar white matter was elevated and measurement of COX 1 and COX 2 proteins showed a corresponding increase although, this was only significant for COX 1 (Fig. 3c and d). As discussed above, complex IV activity and protein levels may be elevated by oxidative stress^45,46^. Correlation between complex activity and the expression levels of protein components did not extend to the other ETC complexes investigated as has been shown by other investigators^52,53^. Despite the observed increase in complex I activity in MSA cerebellar white matter NDUFB8, a nuclear encoded supernumerary subunit located on the inner mitochondrial membrane, showed no changes in protein level (Fig. 2b). This may reflect the observation that this subunit does not influence the complex activity as it does not contain an active domain^54^. Interestingly, the GRIM19/NDUFA13 subunit showed an increase in expression in the MSA cerebellar white matter and PD occipital white matter which correlates with the increased complex I activity (Fig. 2c). This subunit is required for electron transfer activity of complex I and is thought to be involved in the interferon/retinoic acid-mediated cell death^55–57^. Furthermore, this subunit has been used as a marker for complex activity in a study looking at complex I deficient patients^58^. A slight increase in GRIM19 expression was observed in MSA occipital white matter which does follow the upward trend in enzyme activity (Figs 2c and 1a). This could indicate early impairment of mitochondrial function in this brain region which is minimally affected by MSA pathology as visualised by cellular inclusions of aggregated α-synuclein. To assess complex II we measured levels of the subunits SDHA and SDHB. SDHA showed an increase in expression in MSA cerebellum compared with controls despite the decrease in activity of complex II/III (Fig. 2d). UQCRC2, a component of complex III showed no change in expression in MSA (Fig. 3a). Interestingly the mitochondrial encoded complex III subunit, cytochrome b (cyto b) demonstrated increased levels in MSA cerebellar white matter and PD occipital white matter (Fig. 3b). Although complex II/III showed reduced activity in both these areas, the increase in cytochrome b expression could be a reflection of its anti-oxidant activation^59,60^. In addition to the influence of CoQ10 reduction in MSA a number of other mechanisms may influence protein function and may be important in regulation of activity of ETC complexes. For example, complex II activity is modulated by post-translational phosphorylation and acetylation^61^. SDHA is phosphorylated in mammalian cells and, like acetylation, this post-translational modification can attenuate complex activity^62^. Moreover, SDH catalytic activity can also be controlled by Krebs cycle intermediates including oxaloacetate, which is a potent inhibitor. The enzymatic status of complex II thus appears to be influenced by factors which are independent of protein expression providing an explanation for the discrepancies in protein expression and activity we observed. Determining the acetylation and phosphorylation status of complex II components would be of interest, however, specific antibodies suitable for immunoblotting are not currently available. Investigation of protein levels of additional mitochondrial ETC complex components may also be informative.

Mitochondrial dysfunction is well described in PD, the most frequent α-synucleinopathy, and PD cases with Braak stage 6 α-synuclein pathology were therefore used as a disease control for this study^63–65^. α-Synuclein pathology in PD is largely restricted to the grey matter and each of the regions we examined in this study typically show little pathology, although, the occipital lobe may be affected in Braak stage 6 disease (Supplementary Fig. 1)^66^. In keeping with this, we found no changes in ETC complex activity or in protein expression of the components examined in the cerebellar white matter in PD compared with control. In the occipital lobe we observed increased complex I and decreased complex II/III activity in PD, mirroring the changes in the cerebellum of MSA and suggesting a common mechanism of tissue damage in these two diseases (Fig. 1a and b). In contrast to our findings, previous studies have demonstrated reduced complex I activity in PD but these have not included analysis of the occipital lobe or enrichment for white matter^49,52,53,67^. In one study of PD in which neuronal depletion of complex I in several brain regions was implicated from immunohistochemical analysis of the component NDUFB8, the immunohistochemical result in the cerebellum was not replicated in immunoblotting or when complex I activity was measured. This concurs with our finding that complex I activity in the cerebellum is unchanged in PD. It also emphasises that results may vary when different methods are compared in brain tissue^49^. Other studies have shown conflicting results when measuring ETC complex activities in PD platelets, lymphocytes, substantia nigra, frontal cortex and muscle^68^. Some report a reduction in activity and others found no changes in both complex II^50,69^ and complex III independently^70–72^. Decreased complex II/III activity has been observed in cortical regions in PD with dementia^52^. It has been shown using proton Magnetic Resonance (MR) spectroscopy that there are increased levels of lactate in the occipital lobe of PD patients compared to controls implicating altered mitochondrial function in this region in addition to nigrostriatal pathways and supporting our finding of impaired mitochondrial function in the occipital lobe in PD^73^.

In conclusion, the results of this study support the hypothesis that ETC dysfunction may be important in the pathogenesis of MSA. The decrease in the cerebellar ETC complex II/III activity may result from the deficit in CoQ10 levels previously described^20,21^. Research into MSA has primarily focused on oligodendroglial and neuronal dysfunction secondary to accumulation of aggregated α-synuclein, leading to microglial activation, neuroinflammation and oxidative stress^2,74,75^. Altered activity of the ETC complexes in the cerebellar white matter with preservation in the occipital lobe strongly supports a role for oligodendroglial mitochondrial dysfunction in the pathogenesis of MSA and the parallels with PD suggest a common disease mechanism in α-synucleinopathies. Whether mitochondrial dysfunction is a primary driver of disease in MSA or is secondary to other pathological processes remains to be determined.

## Materials and Methods

### Samples

The brains were donated to the Queen Square Brain Bank for Neurological Disorders, UCL Institute of Neurology using ethically approved protocols and stored for research under a licence issued by the Human Tissue Authority. The brain was routinely hemi-dissected in the sagital plane and half was sliced and flash frozen. Samples of cerebellar and occipital white matter were dissected from the frozen brain tissue and then homogenised for the mitochondrial assays, as well as western blotting. These samples are enriched for white matter as this study was designed to characterise changes in this tissue which is affected by GCI pathology in MSA. Corresponding brain regions from flash frozen tissue were also cut to provide 8 µm thick sections for the COX/SDH histochemistry. The cerebellar white matter, a region with large numbers of α-synuclein positive GCIs^4,35^ was compared with the occipital white matter where GCIs are sparse in MSA^36,37^. Both regions are minimally affected in PD in which they may contain sparse Lewy neurites (Supplementary Fig. 1).

Mixed MSA cases with short post-mortem delay were selected in order to minimise the potential biological changes after death. Neurologically normal cases were used as controls in addition to PD cases with advanced, Braak stage 6, pathology which acted as an α-synucleinopathy disease control. The groups were matched as closely as possible for age at death and gender, details are provided in Table 2.

**Table 2:** Demographics of the case cohort [s.e.m.; Standard error of the mean].

Case group	Number	Age (years) ± s.e.m.	Gender (Male/Female)	Post-mortem delay (hours: minutes ± s.e.m.)	Brain regions
Control	10	86 ± 2	5/5	67:34 ± 10	Cerebellar white matter and occipital white matter
MSA	10	64 ± 2	6/7	38:28 ± 5	
PD (Braak stage 6)	10	80 ± 2	5/5	25:20 ± 5	

### Determination of ETC enzyme activities

Both the cerebellar white matter and occipital white matter were sampled from 10 flash frozen neurologically normal controls, mixed MSA and PD cases (Table 2). Each brain region was homogenised and the activities of ETC complex I (NADH: ubiquinone reductase), II/III (succinate: cytochrome c reductase) and IV (cytochrome c oxidase) together with CS were assayed spectrophotometrically at 30 °C as previously described^76^. CS is routinely used as a marker of mitochondrial biomass. It is the rate- limiting step in the tricarboxylic acid cycle (TCA) cycle and its activity is therefore not dependent on mitochondrial-encoded proteins^77^. The CS activity was measured and normalised to total protein content which was calculated using bicinchoninic acid (BCA) assay (Thermo Scientific, Massachusetts, US)^78^.

### Protein homogenisation and western blotting

Flash frozen tissue weighing ~0.5 g was homogenised in high- salt lysis buffer (50 mM Tris HCL pH 7.4, 175 mM NaCL, 1% Triton X with protease and phosphatase inhibitor tablets (Roche, Basel, Switzerland), 1 tablet per 50 ml). The tissue was homogenised in a volume 5 times of its weight using a glass dounce. The lysate was centrifuged at 1000 × g for 5 minutes at 4 °C to remove cell debris. The supernatant was removed and stored at −80 °C. A protein determination assay (BCA, Thermo Scientific Massachusetts, US) was carried out where the lysate was diluted in reducing agent (Invitrogen) and in lithium dodecyl sulfate (LDS) sample buffer (Invitrogen, California, US). For mitochondrial complexes it is recommended not to boil the samples as this will reduce the signal of several bands. For the SDS/PAGE electrophoresis, 10 µg of each sample was loaded into a 4–12% Bis/Tris 1.0 mm gel and run in MES SDS running buffer (Invitrogen, California, US) containing antioxidant (Invitrogen, California, US) at 120 V. The gels were then transferred to nitrocellulose membrane (GE Healthcare, Illinois, US) using XCell blot Invitrogen equipment in transfer buffer containing 10% methanol for 1 hour and 30 minutes. The membrane was then blocked in 5% semi-dry powdered milk/PBS for 1 hour at RT on a shaker. The membranes were incubated with in a primary antibody diluted in 2% BSA/PBS- 0.1%Tween (PBS-T) over night at 4 °C on the shaker (See Table 3 for antibody information). The following day, the membrane was washed in PBS-T and incubated with a LiCOR 680/880 secondary antibody for an hour on the shaker at 4 °C. The membranes were then washed three times with PBS-T, followed by a fourth wash in PBS before developing the blots on the LiCOR Odyssey machine. Two loading controls were used, β-actin and citrate synthase. For technical quality reasons case numbers analysed varied between proteins and regions examined (n = 3–10). Each sample was run in triplicate over different gels, each containing control, MSA and PD samples within one gel. For the analysis, each band intensity was analysed using Fiji Image J software^79^ across the triplicates. Each complex subunit was normalised to citrate synthase and then the triplicate values were averaged. These values are represented as bar graphs. Each gel is presented in full for each western blot in Supplementary Figs 2–7.

**Table 3:** Antibodies used for determining the mitochondrial ETC complex protein expression.

Antibody	Abcam (Cambridge, UK) reference	Species	Dilution	Complexes detected and molecular weight
NDUFB8	ab123545	Mouse	1 in 250	NDUFB8- 22 kDa
GRIM19	ab110240	Mouse	1 in 1000	GRIM19- 17 kDa
MitoBiogenesis™ Western blot Cocktail	ab123545	Mouse	1 in 500	COX 1 (cIV)- 35 kDa
				SDHA (cII) - 70 kDa
				β actin - 42 kDa
Cytochrome b	Milliopore MAB52036	Mouse	1 in 1000	Cytochrome c- 28 kDa
Citrate synthase	ab96600	Rabbit	1 in 5000	52 kDa
Total OXPHOS Human WB Antibody Cocktail	ab110411	Mouse	1 in 1000	UQCR2 (c III)- 48 kDa
				SDHB (cII)- 29 kDa
				COX 2 (cIV)- 22 kDa

### COX/SDH assay

Frozen sections (8 µm) from the cerebellar and occipital white matter of control, PD and MSA cases (n = 3 for each group, 2 sections per case) were cut and mounted on glass slides. For the succinic dehydrogenase (SDH) assay, the solutions and methods were validated and performed according to the method described by Nachlas *et al*. (1957)^80^. For the COX assay, the solutions and methods were validated according to published methods^81^. Finally the sequential COX/SDH used both, COX incubating medium and the SDH staining solution. The sections were removed from −80 °C to acclimatise to RT and then incubated with COX incubating medium at 37 °C for 2 hours. Following this, the sections were drained and rinsed in deionized water before applying SDH staining solution and incubating at 37 °C for 2 hours. The sections were washed in distilled water and mounted using warmed glycerine jelly (Sigma, Missouri, US).

Stained sections were imaged on the Olympus Virtual Slide Microscope VS120 at a 40x magnification. Four regions of white matter were randomly selected for each case (2 sections per case) using the Image analysis Olympus software, by an observer blind to the diagnostic category of the cases. In these regions the number of blue stained cells, representing the COX deficient cells was counted. The brown, COX positive cells, were also counted to establish a total cell count. The mean count was calculated for each case and the percentage of COX deficient cells was established.

### Equipment and settings

Western blot images were acquired using LI-COR Odyssey Infrared Imaging System Model 9120 which involves a 2 colour detection method using IRDye 800CW or 680RD secondary antibodies. The membranes are imaged at an intensity of 5 ± 2 depending on the antibody using both channels. These images are then converted to a black/white image using Grayscale tool in LI-COR imaging software. This image is converted to a TIFF file so it can be analysed in Fiji Image J software package. A rectangle box is drawn around the band where the pixel intensity is calculated. The pixel intensity of the protein of interest is then divided by the corresponding citrate synthase band intensity so to normalise for protein concentration.

### Graphs and Statistics

All figures with bar graphs were generated in Graphpad and represent the average value ± standard error of mean (s.e.m.). For all figures, an unpaired t test using Mann-Whitney post hoc comparing MSA and PD to control was carried out. Significance was set at p < 0.05 (*), p < 0.01 (**) and p < 0.001 (***). Individual p values are specified in Table 1.

## Ethics Approval and Consent to Participate

Written informed consent was obtained from all participants. Tissue stored in the QSBB is under license 12198 from the Human Tissue Authority and has been donated for research according to protocols approved by the NRES Committee London- Central.

## Supplementary information

Supplementary information

## Data Availability

The datasets used and analysed during the current study are available from the corresponding author on reasonable request.
