# Supplementary material for: Cerebral mitochondrial electron transport chain dysfunction in multiple system atrophy and Parkinson’s disease
Source: Sci Rep. 2019 Apr 25;9:6559. doi: 10.1038/s41598-019-42902-7 (PMC6484105; doi:10.1038/s41598-019-42902-7)
Supplement: Supplementary file 1 — Supplementary information [file 41598_2019_42902_MOESM1_ESM.pdf]

# Cerebral mitochondrial electron transport chain dysfunction in multiple system atrophy and Parkinson's disease

***Sandrine C Foti*[1,2], *Iain Hargreaves*[3,4], *Stephanie Carrington*[3], *Aoife P Kiely*[1], *Henry Houlden*[3] *and Janice L Holton.*[1,*]**

1. *Queen Square Brain Bank for Neurological Disorders, Department of Clinical and Movement Neurosciences, UCL Queen Square Institute of Neurology, University College London, London, UK*
2. *Department of Neurodegenerative Diseases, UCL Queen Square Institute of Neurology, University College London, London, UK*
3. *UCL Institute of Neurology, National Hospital for Neurology and Neurosurgery, London, WC1N 3BG, United Kingdom*
4. *Liverpool John Moores University, School of Pharmacy and Biomedical Sciences, Liverpool, L3 3AF, United Kingdom*

*Janice.holton@ucl.ac.uk

__Supplementary Information__

**Supplementary Data**

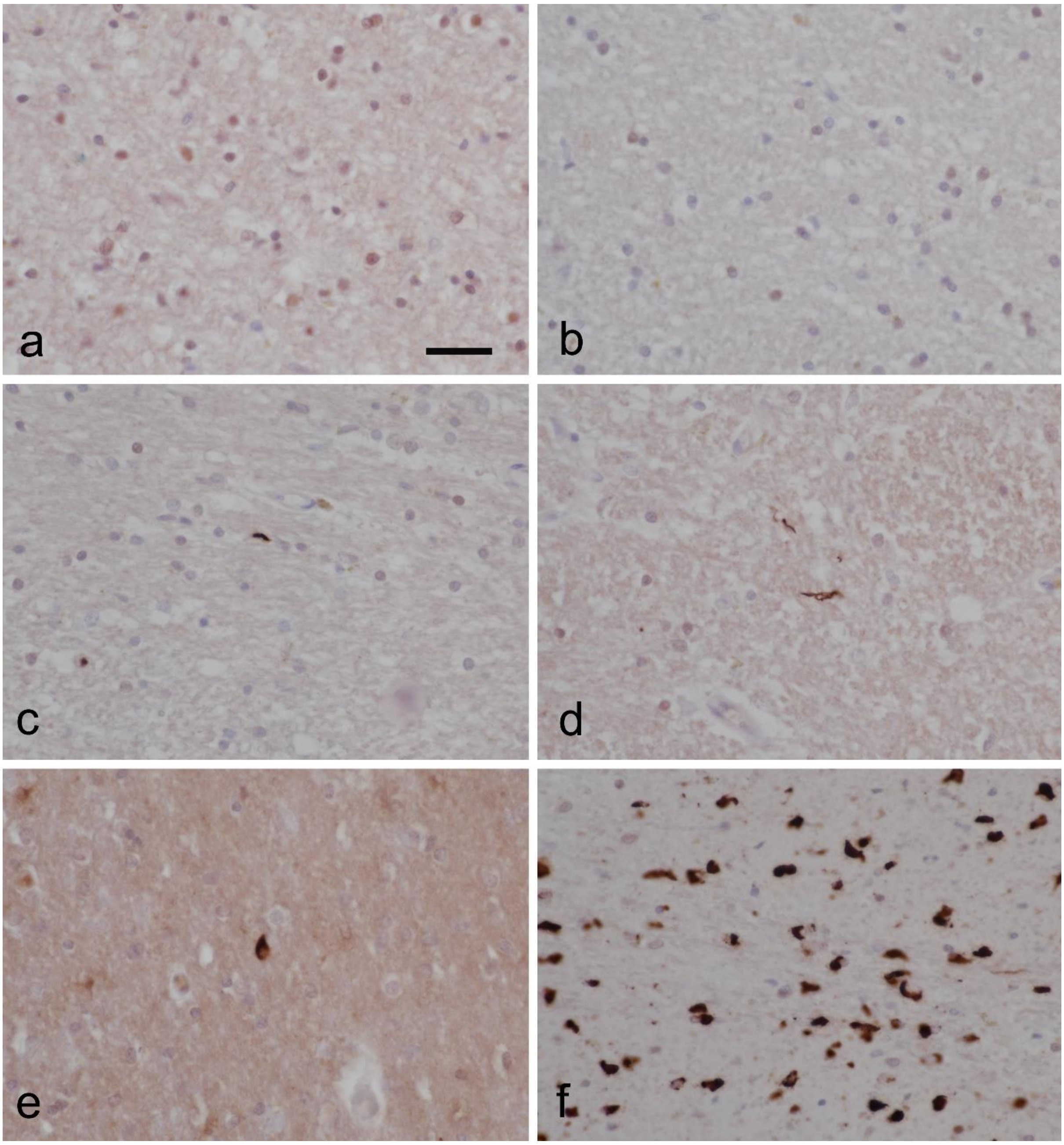


***Supplementary Figure 1:*** *Immunohistochemical staining for α-synuclein.* Immunohistochemical staining for α-synuclein is illustrated in representative control (a, b), PD (c, d) and MSA (e, f) cases. In the occipital white matter (a, c, e) there are no pathological aggregates in the control (a), Lewy neurites are rare in PD and there are infrequent GCI in MSA (e). Similarly, in the cerebellar white matter (b, d, f) there are no pathological aggregates in the control (b) and Lewy neurites are sparse in PD (d). In contrast there are numerous GCI in the cerebellar white matter in multiple system atrophy (f). Bar in (a) represents 30μm in all images.

**The original full length immunoblots related to Figures 2d and 3c**

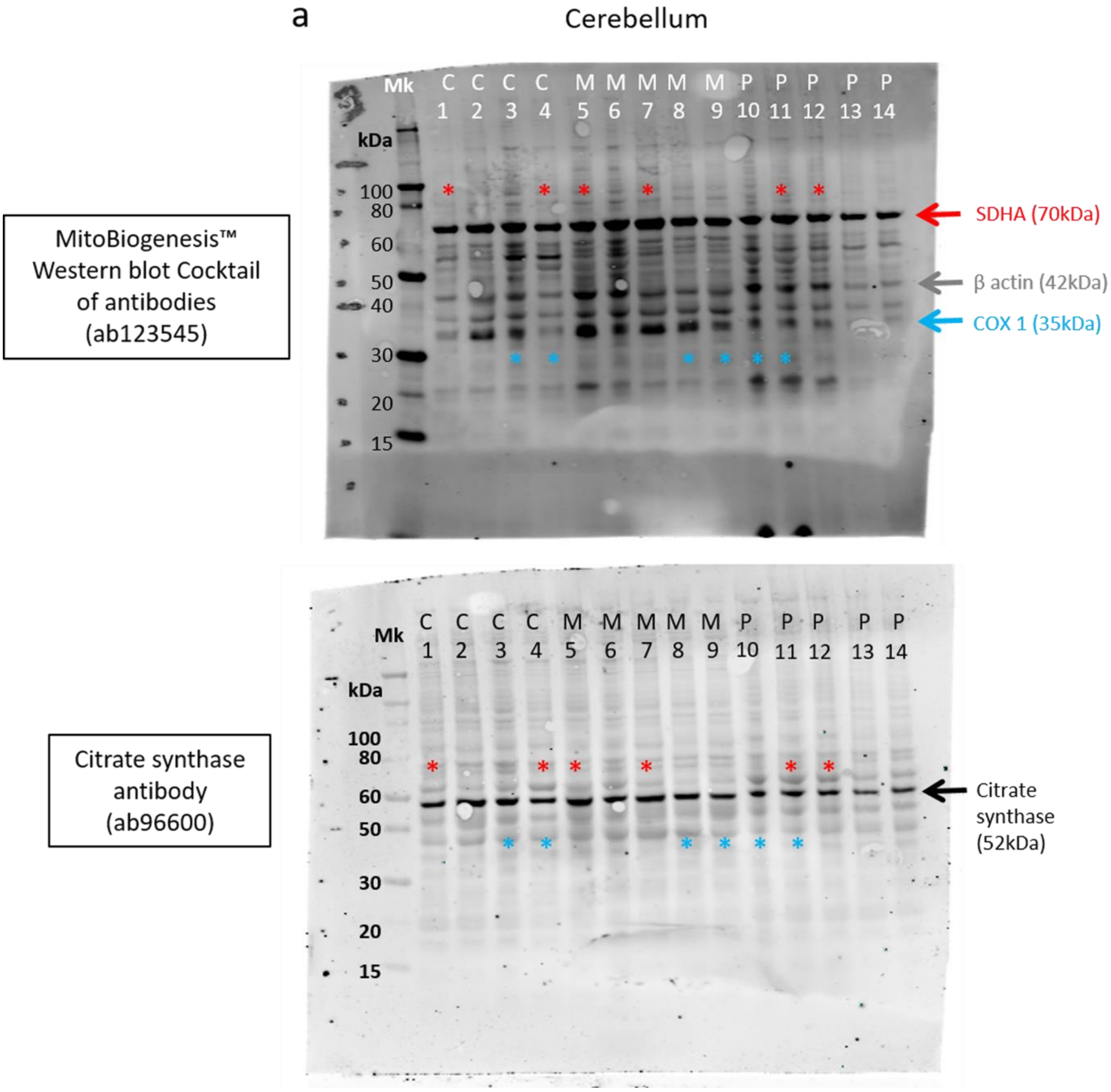

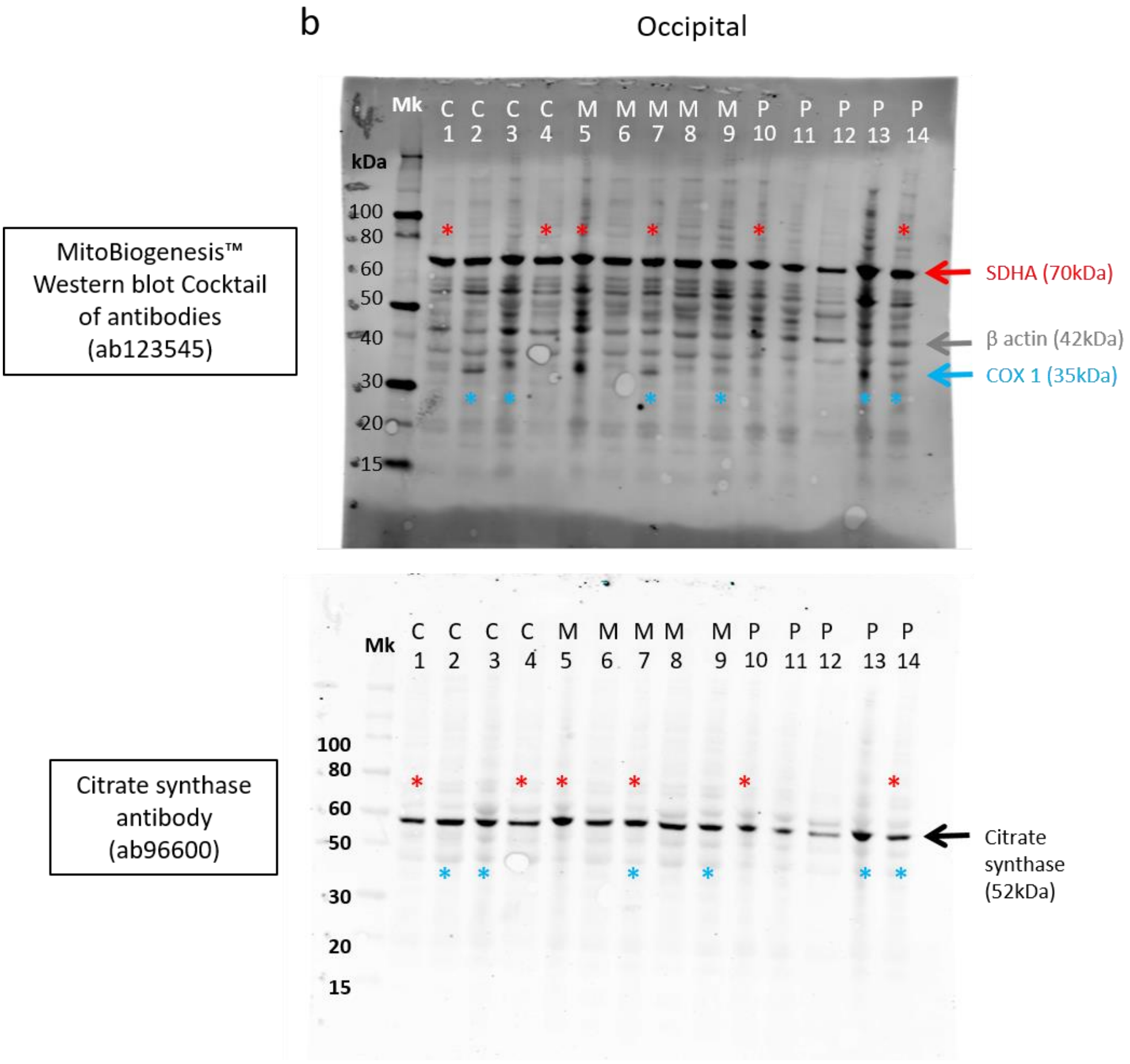


***Supplementary Figure 2:*** *Western blots probed for SDHA and COX 1 subunits from ETC complexes in control, MSA and PD samples*. A MitoBiogenesis™ Western blot cocktail of antibodies was used which recognise subunit SDHA of complex II, COX 1 subunit from complex IV as well as B actin. Cerebellar white matter (a) and occipital white matter (b) were extracted from control (C), MSA (M) and PD (P) cases and separated using SDS-PAGE electrophoresis. These blots were run in triplicate where the band intensities were measured using Fiji image J analysis software. Each band was normalised to the band intensity of the loading control, citrate synthase. These values of each sample across the triplicate blots were averaged. The * represent the bands chosen as the representative blots (red=SDHA, blue=COX 1) shown in Figures 2c and 3b. The numbers represent the sample number. [Mk; markers, kDa; kiladaltons; SDHA; succinate dehydrogenase complex flavoprotein subunit A, COX 1; cytochrome c oxidase 1]

**The original full length immunoblots related to Figures 2b**

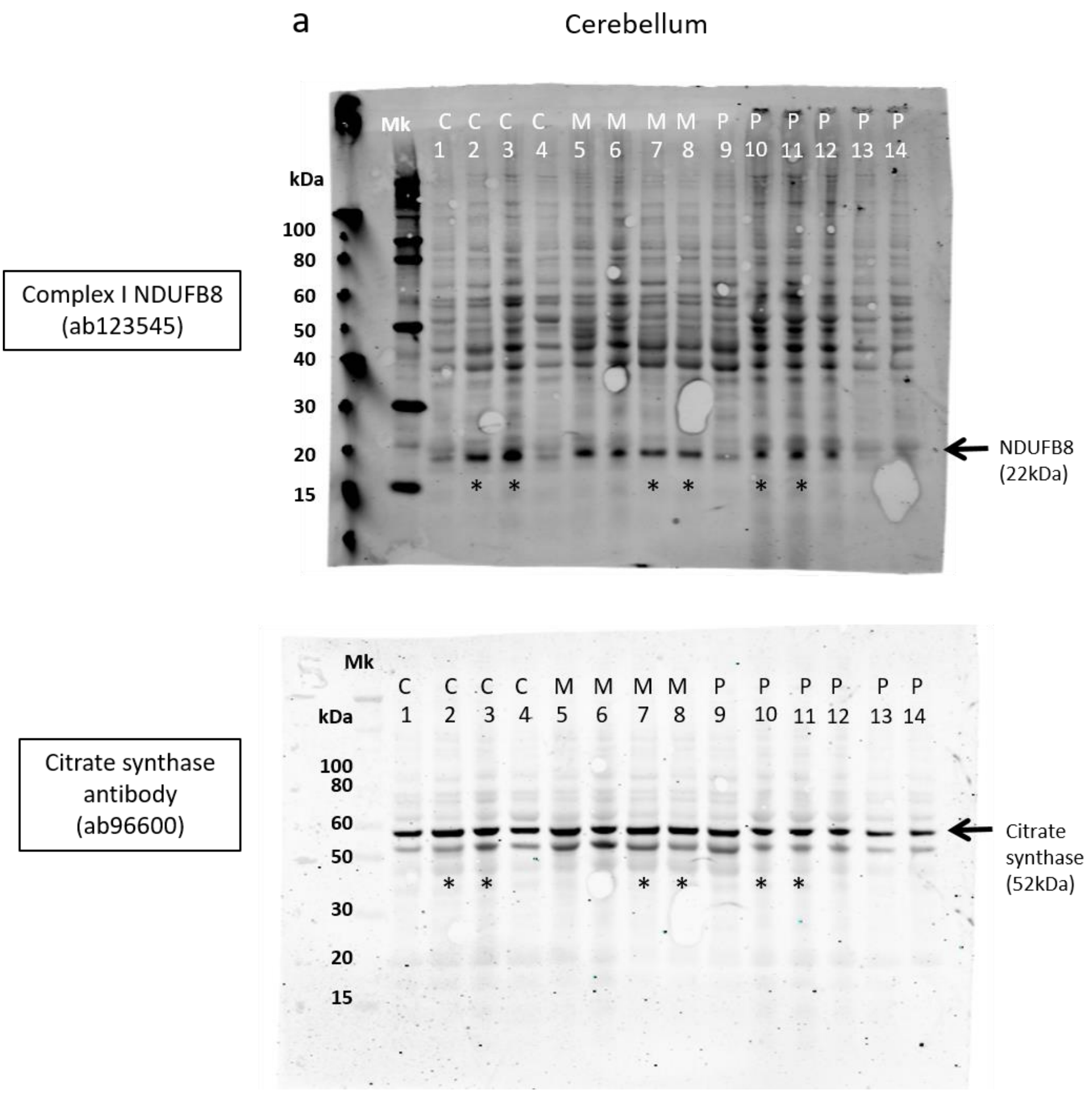

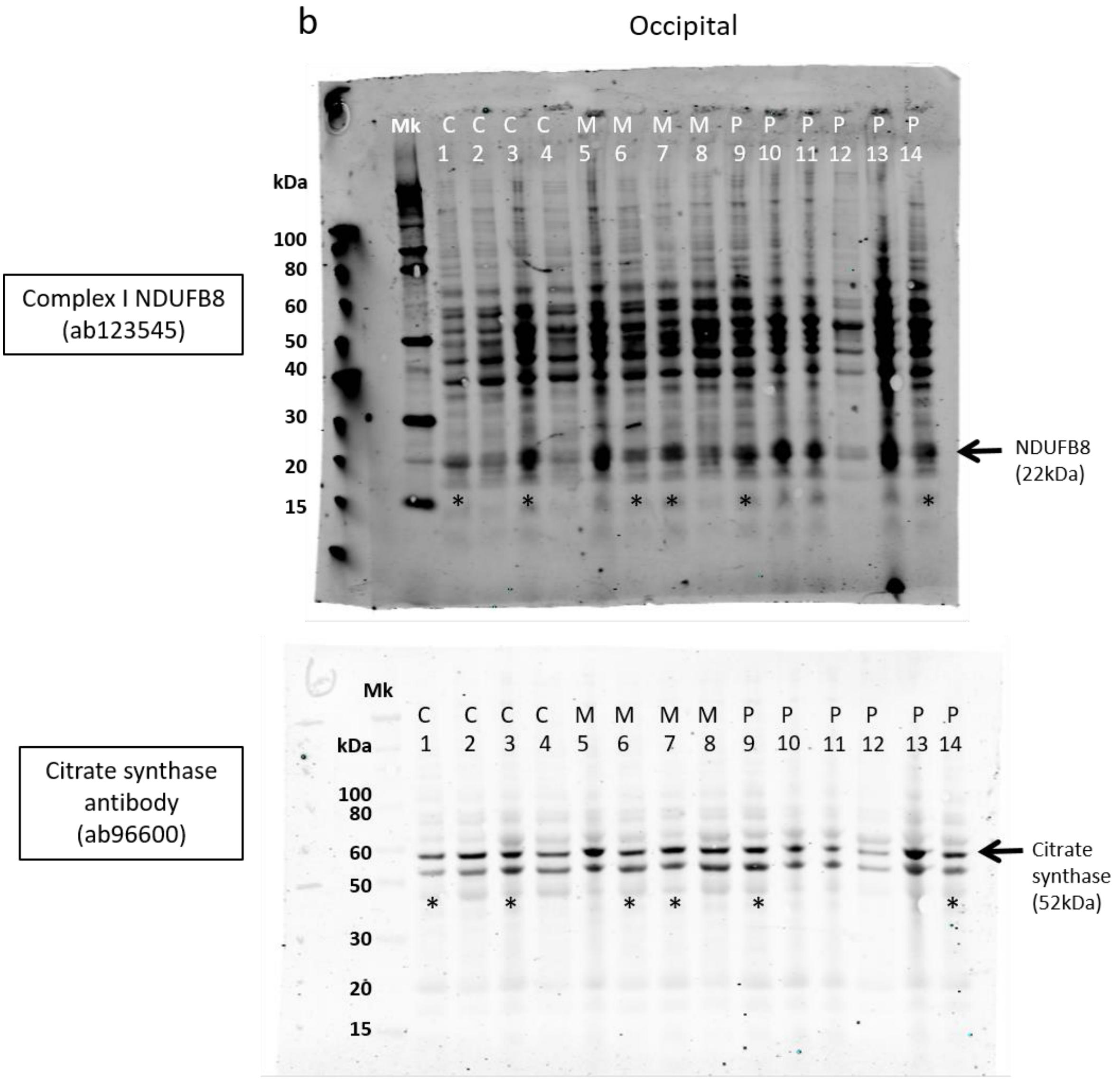


***Supplementary Figure 3:*** *Western blots probed for NDUFB8 subunit from complex I subunit in control, MSA and PD samples*. Cerebellar white matter (a) and occipital white matter (b) were extracted from control (C), MSA (M) and PD (P) cases and separated using SDS-PAGE electrophoresis. These blots were run in triplicate where the band intensities were measured using Fiji image J analysis software. Each band was normalised to the band intensity of the loading control, citrate synthase. These values of each sample across the triplicate blots were averaged. The * represent the bands chosen as the representative blots shown in Figures 2b. The numbers represent the sample number. B actin was not analysed as this antibody. [Mk; markers, kDa; kilodaltons; NDUFB8; NADH dehydrogenase [ubiquinone] 1 beta subcomplex subunit 8]

**The original full length immunoblots related to Figures 2e, 3a and 3d**

a

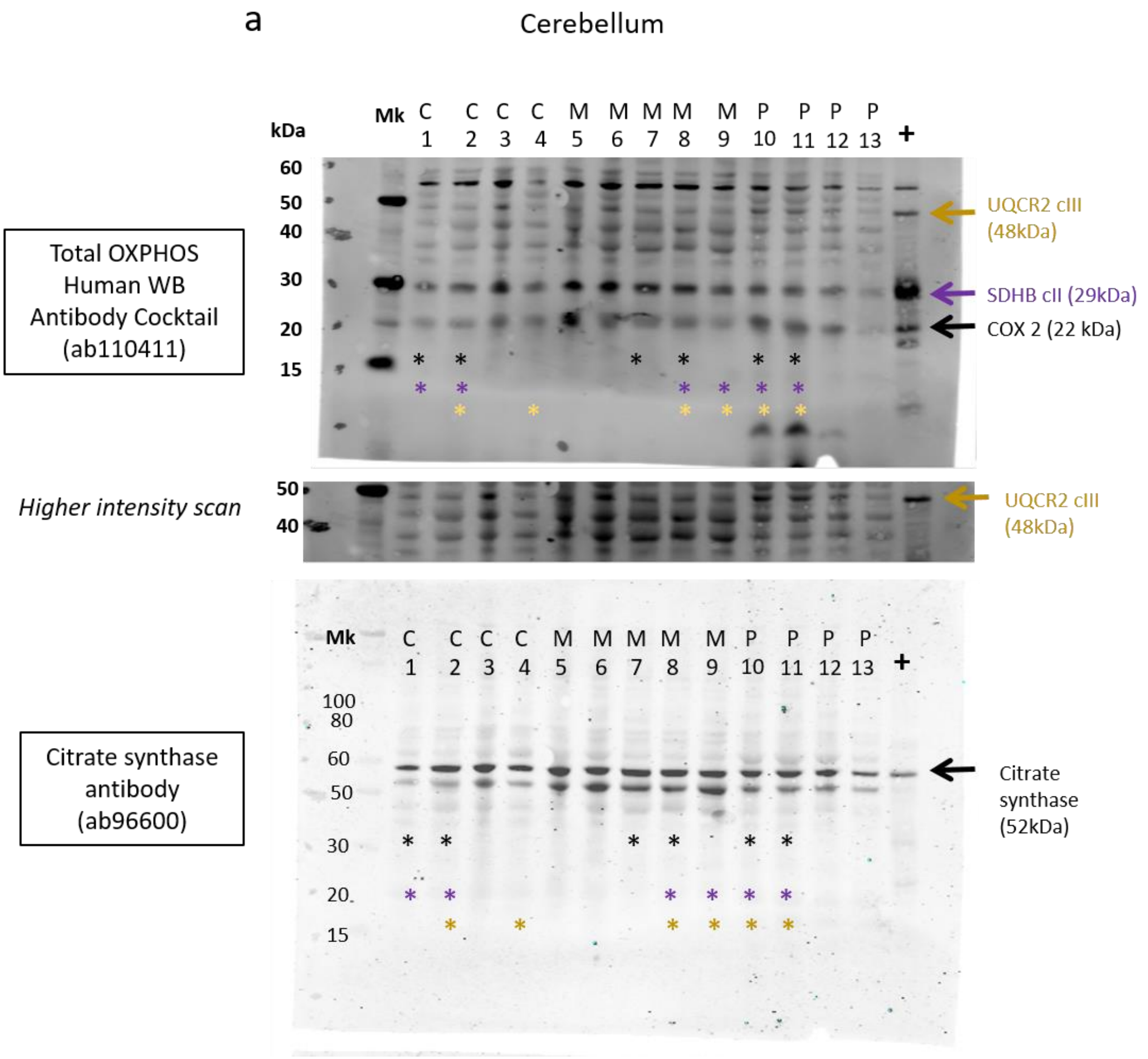

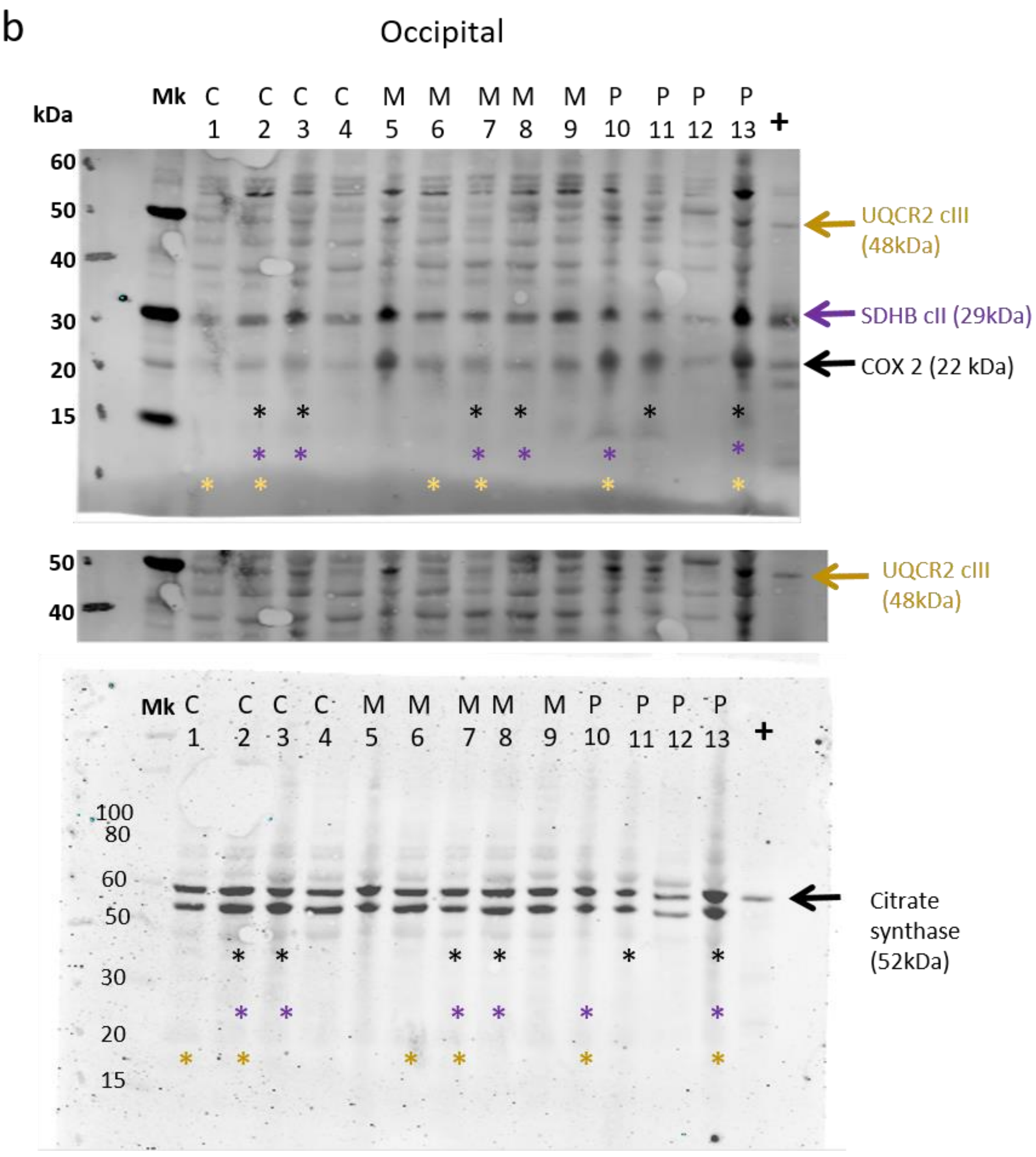


***Supplementary Figure 4:*** *Western blots probed for UQCR2, SDHB and COX 2 subunits from ETC complexes in control, MSA and PD samples*. A Total OXPHOS Human WB antibody cocktail of antibodies was used which recognise subunit SDHB of complex II, COX 2 subunit from complex IV and UQCR2 from complex III. Cerebellar white matter (a) and occipital white matter (b) were extracted from control (C), MSA (M) and PD (P) cases and separated using SDS-PAGE electrophoresis. These blots were run in triplicate where the band intensities were measured using Fiji image J analysis software. Each band was normalised to the band intensity of the loading control, citrate synthase. These values of each sample across the triplicate blots were averaged. The * represent the bands chosen as the representative blots (yellow=UQCR2, purple=SDHB, black=COX 2) shown in Figures 2d, 3a and 3c. The numbers represent the sample number. An image at a higher intensity scan of the same blot is illustrated below where UQCR2 band was used in the presentative blot in Figure 3a. [+; positive control, Mk; markers, kDa; kilodaltons; UQCR2; ubiquinol-cytochrome c reductase core protein 2 SDH2, COX 2; cytochrome c oxidase 2]

**The original full length immunoblots related to Figure 2a**

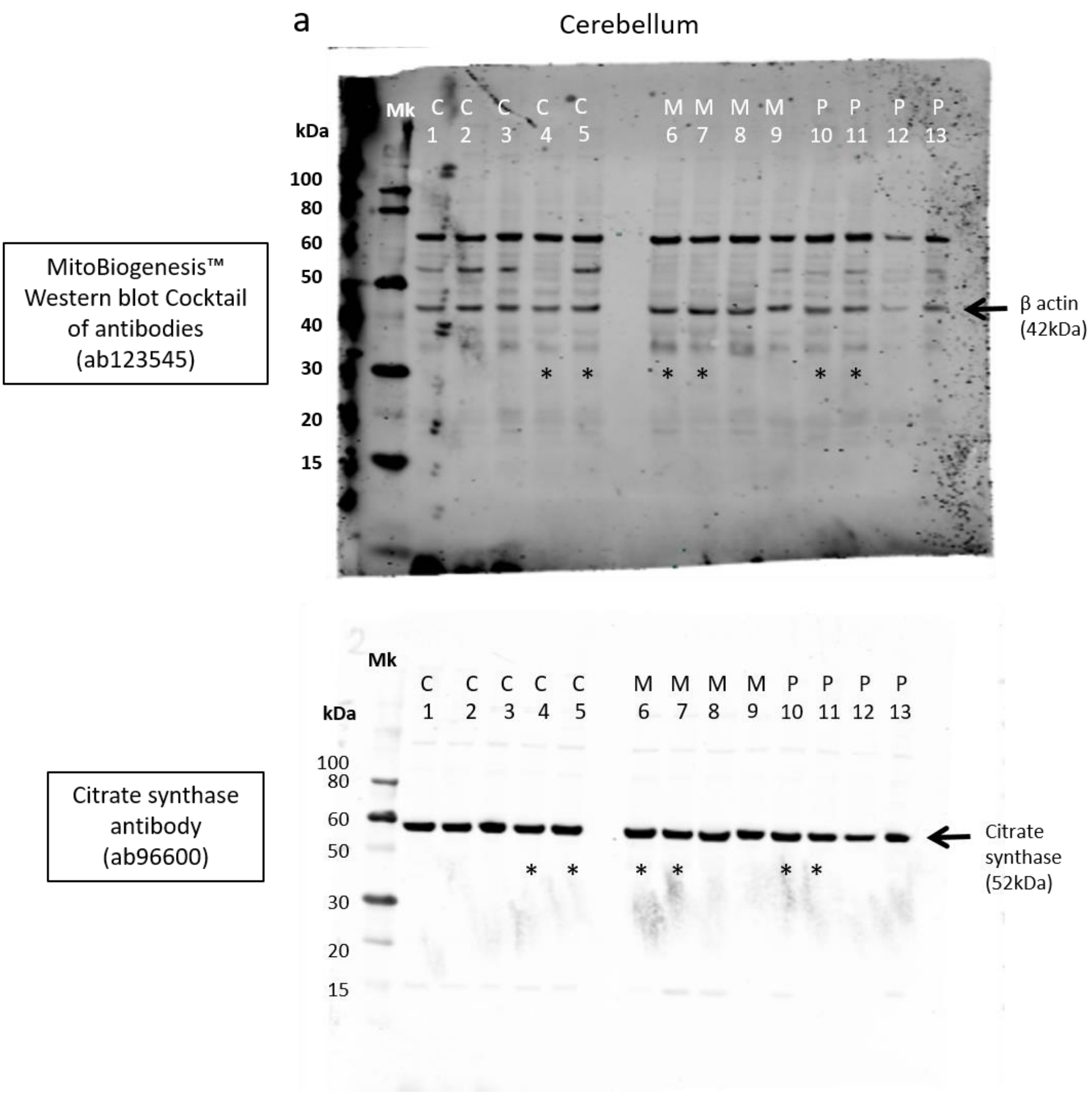

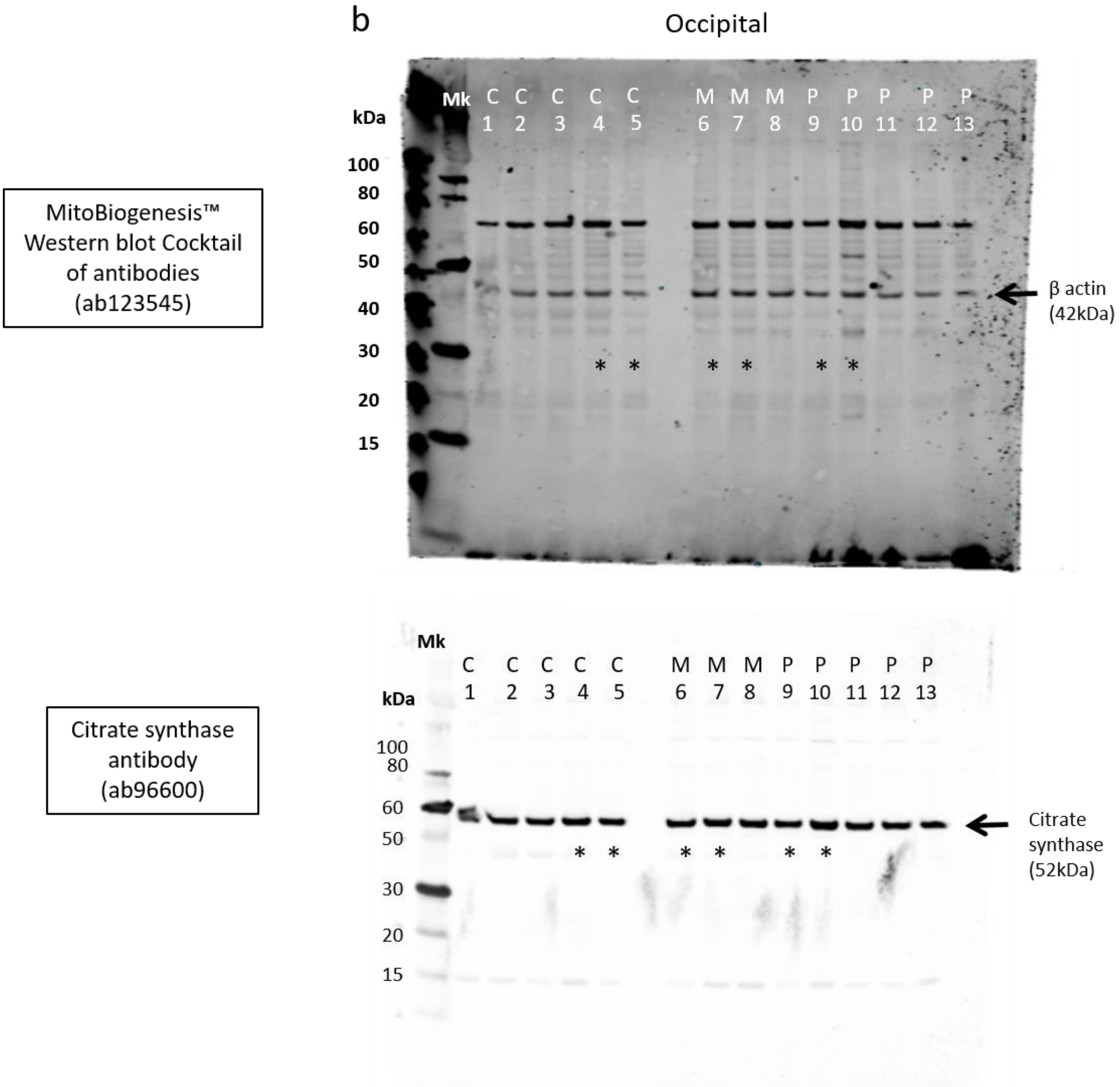


***Supplementary Figure 5:*** *Western blots probed for β actin in control, MSA and PD samples*. A MitoBiogenesis™ Western blot cocktail of antibodies was used which recognises β actin. Cerebellar white matter (a) and occipital white matter (b) were extracted from control (C), MSA (M) and PD (P) cases and separated using SDS-PAGE electrophoresis. These blots were run in triplicate where the band intensities were measured using Fiji image J analysis software. Each band was normalised to the band intensity of the loading control, citrate synthase. These values of each sample across the triplicate blots were averaged. The * represent the bands chosen as the representative blots shown in Figure 2a. The numbers represent the sample number. [Mk; markers, kDa; kilodaltons;]

**The original full length immunoblots related to Figure 2c**

GRIM19 (ab110240)

Citrate synthase antibody (ab96600)

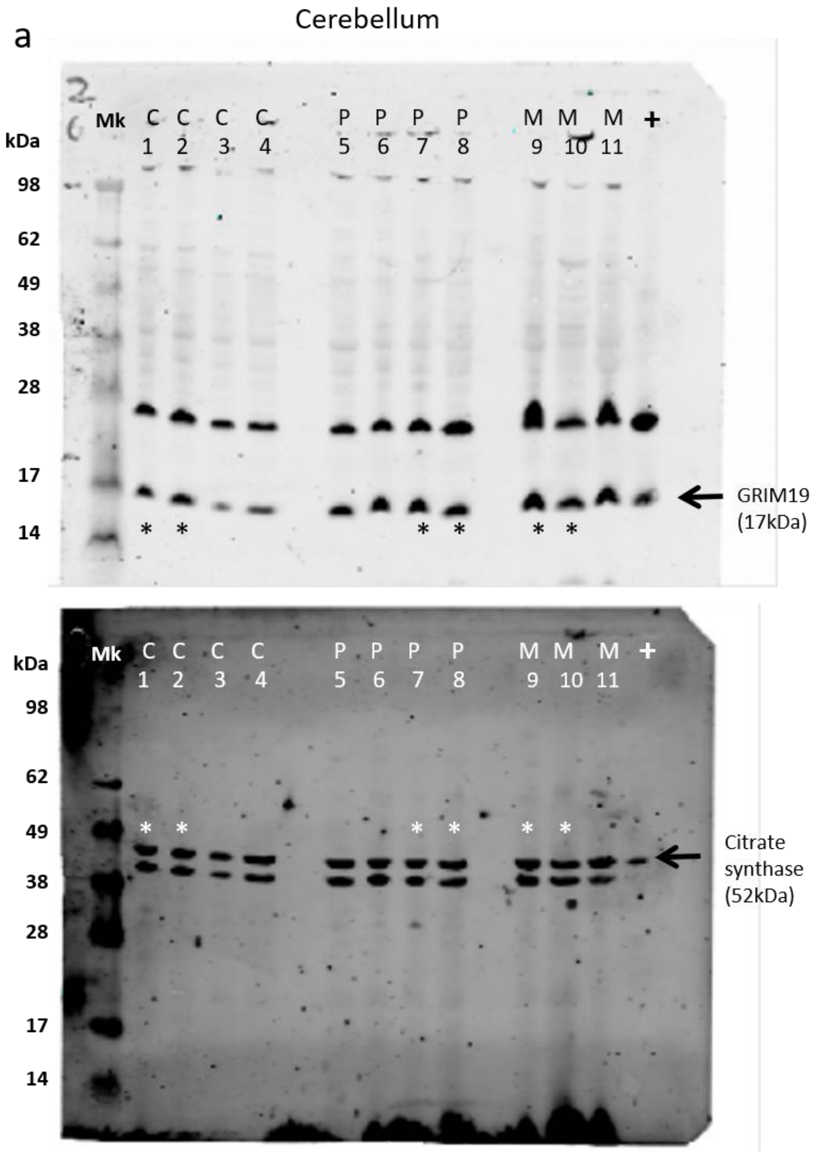

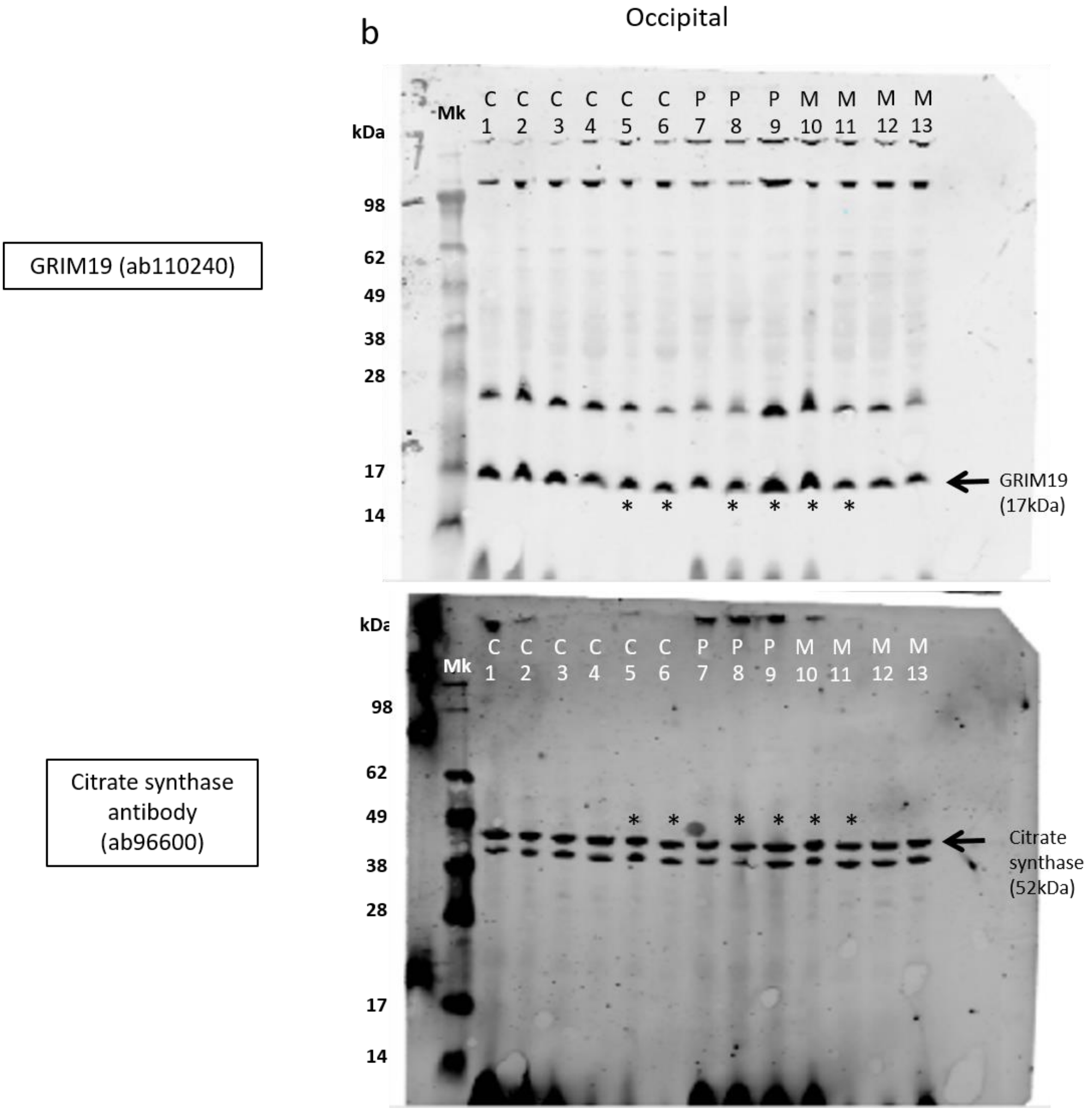


***Supplementary Figure 6:*** *Western blots probed for GRIM19 in control, MSA and PD samples*. An anti-GRIM19 antibody was used which recognises GRIM19 at 17 kDa. Cerebellar white matter (a) and occipital white matter (b) were extracted from control (C), MSA (M) and PD (P) cases and separated using SDS-PAGE electrophoresis. These blots were run in triplicate where the band intensities were measured using Fiji image J analysis software. Each band was normalised to the band intensity of the loading control, citrate synthase. These values of each sample across the triplicate blots were averaged. The * represent the bands chosen as the representative blots shown in Figure 2c. The numbers represent the sample number. [Mk; markers, kDa; kilodaltons; GRIM19; Genes associated with Retinoid–IFN-induced Mortality-19]

**The original full length immunoblots related to Figure 3b**

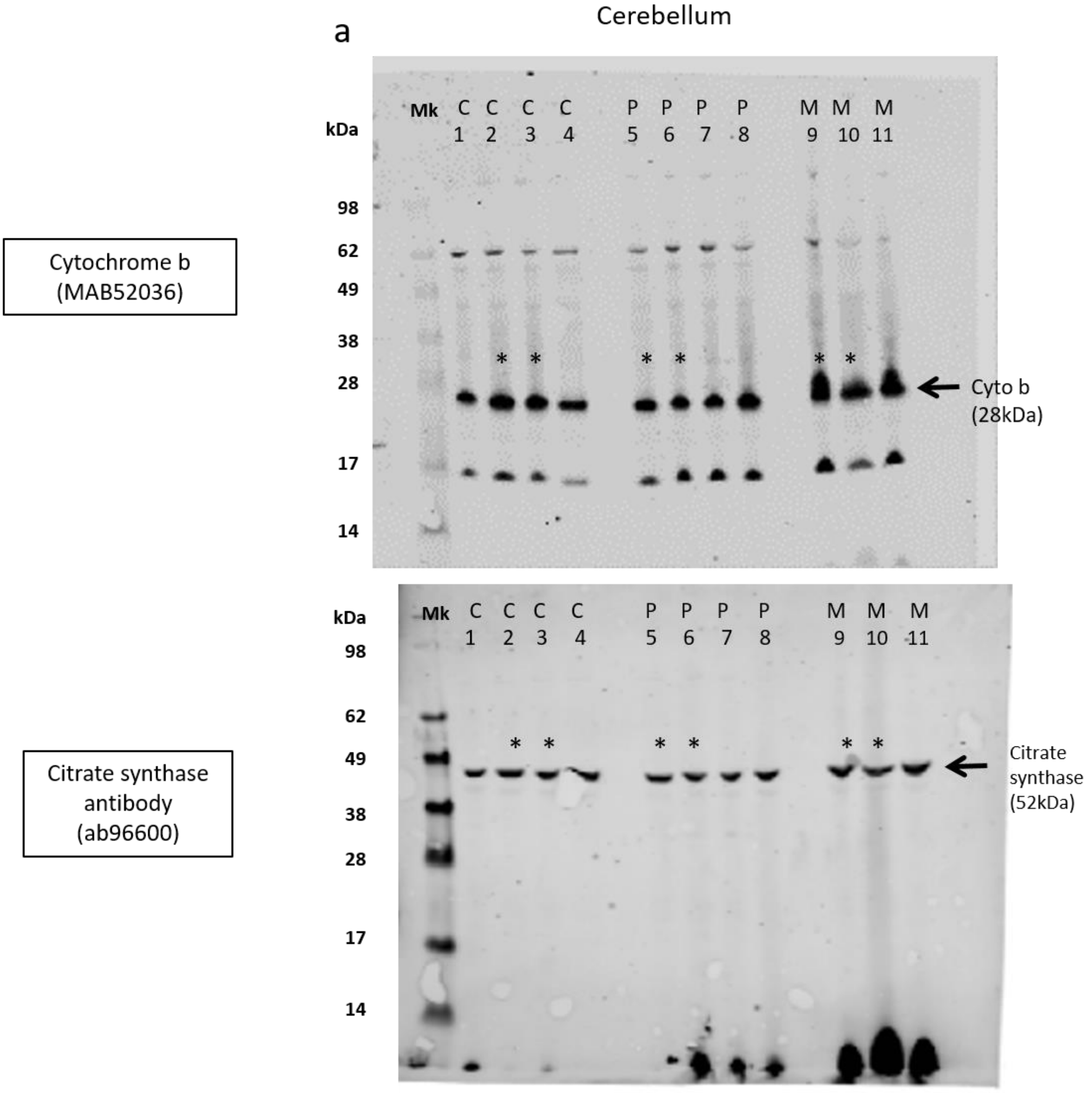

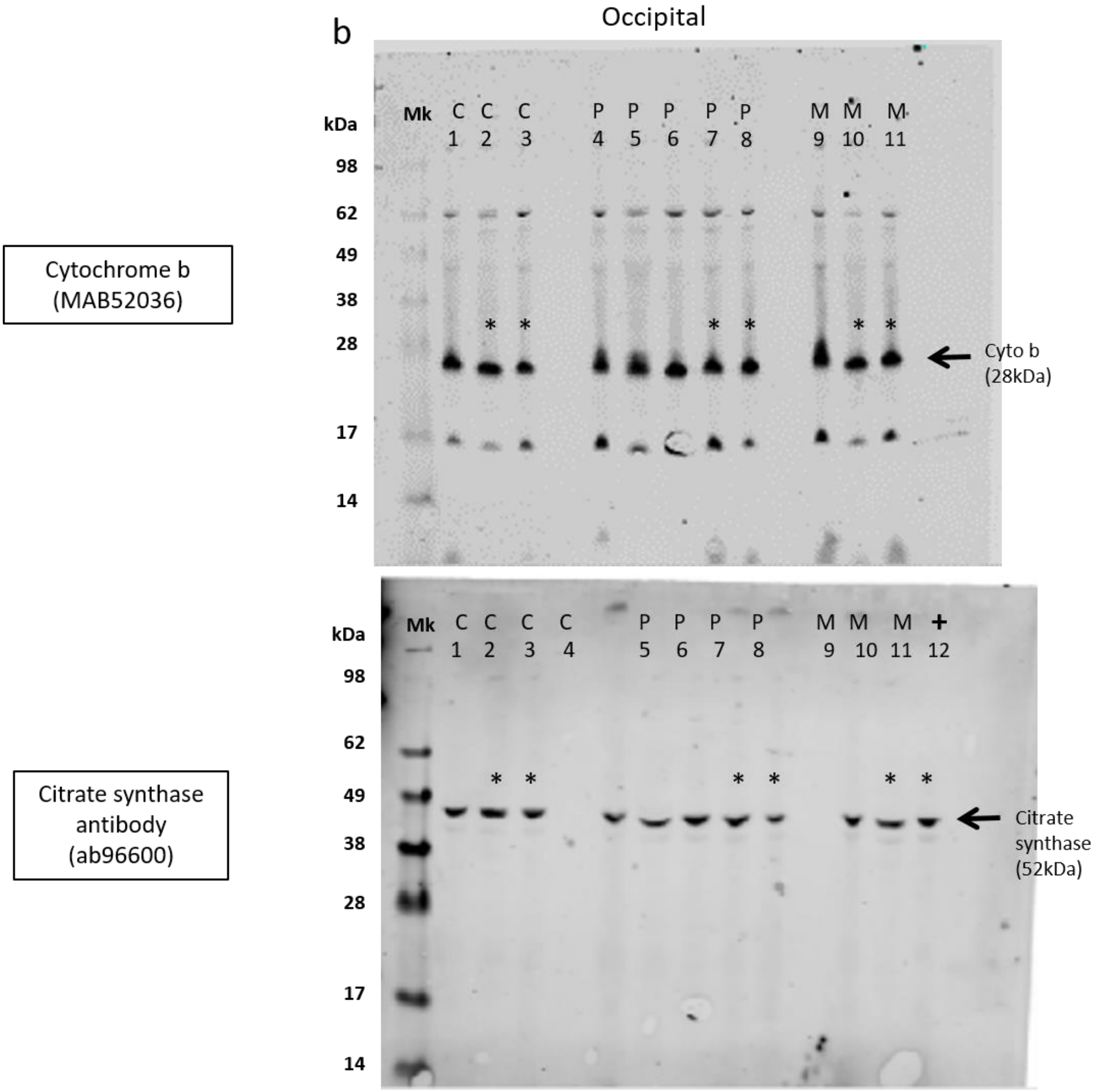


***Supplementary Figure 7:*** *Western blots probed for cytochrome b in control, MSA and PD samples*. An anti- cytochrome b antibody was used which recognises cytochrome b at 28 kDa. Cerebellar white matter (a) and occipital white matter (b) were extracted from control (C), MSA (M) and PD (P) cases and separated using SDS-PAGE electrophoresis. These blots were run in triplicate where the band intensities were measured using Fiji image J analysis software. Each band was normalised to the band intensity of the loading control, citrate synthase. These values of each sample across the triplicate blots were averaged. The * represent the bands chosen as the representative blots shown in Figure 3b. The numbers represent the sample number. [Mk; markers, kDa; kilodaltons; Cyto b; cytochrome b]
